# Cystatin-c / total cholesterol ratio as a predictor of probable sarcopenia in geriatric population from 12 European countries

**DOI:** 10.1007/s40520-025-03007-6

**Published:** 2025-03-17

**Authors:** Rizwan Qaisar, M. Azhar Hussain, Asima Karim, Firdos Ahmad, Atif Awad, Mohammed Alsaeed, Shaea A. Alkahtani

**Affiliations:** 1 Basic Medical Sciences, College of Medicine, University of Sharjah, Sharjah, United Arab Emirates; 2 Space Medicine Research Group, Research Institute for Medical and Health Sciences, University of Sharjah, Sharjah, United Arab Emirates; 3 Cardiovascular Research Group, Research Institute for Medical and Health Sciences, University of Sharjah, Sharjah, United Arab Emirates; 4 Department of Finance and Economics, College of Business Administration, University of Sharjah, Sharjah, United Arab Emirates; 5 Department of Social Sciences and Business, Roskilde University, Roskilde, Denmark; 6 Department of Biomechanics & Motor Behavior, College of Sport Sciences and Physical Activity, King Saud University, Riyadh, 11451 Saudi Arabia; 7 Exercise Physiology Department, College of Sport Sciences and Physical Activity, King Saud University, PO Box: 1949, Riyadh, 11451 Saudi Arabia

**Keywords:** Cystatin-c, Total cholesterol, Handgrip strength, Sarcopenia, SHARE data

## Abstract

**Background:**

A low handgrip strength (HGS) is associated with several diseases in older adults. However, predictive biomarkers of future low HGS are poorly known. We investigated the prognostic efficacy of the ratio of plasma cystatin-c and total cholesterol (CT ratio) levels for predicting future low HGS in Europe.

**Methods:**

The data was collected from the Survey of Health, Ageing, and Retirement in Europe (SHARE) between 2015 and 2021-22. The study participants were geriatric adults aged 50 or above (*n* = 17,698) from 12 European countries. The baseline data in 2015 included the measurements of CT ratio from participants with normal HGS. The participants who developed low HGS in subsequent years were included. We also investigated the quality of life, difficulties performing daily activities, and several comorbidities in the study population.

**Results:**

In a multivariate-adjusted model, male gender, advancing age, poor quality of life, difficulties performing daily activities, and the presence of depression, high blood pressure, diabetes mellitus, Alzheimer’s disease, and osteoarthritis were associated with a higher risk of developing low HGS. CT ratio exhibited significant prognostic accuracy for low HGS among men and women aged 60–79, irrespective of the quality of life, degree of depression, difficulties performing daily activities, and the presence of comorbidities, including depression, high blood pressure, diabetes mellitus, Alzheimer’s disease, and osteoarthritis.

**Conclusion:**

Collectively, the CT ratio exhibits adequate prognostic accuracy for low HGS, which is not significantly affected by comorbidities and functional limitations. Our findings have clinical and policy implications in timely identifying older adults at risk of developing low HGS.

## Background

Sarcopenia, a progressive skeletal muscle disorder, is characterized by a decline in muscle mass, strength, and function [1]. While often associated with aging, its onset can occur earlier in life. External factors, including physical inactivity, nutritional deficiencies, chronic diseases, and hormonal imbalances, contribute to sarcopenia and can lead to adverse outcomes such as falls, frailty, disability, and increased mortality [2].

The European Working Group on Sarcopenia in Older People (EWGSOP) has identified low muscle strength as a key diagnostic criterion of sarcopenia [3]. The diagnosis is confirmed by assessing low muscle quantity and quality, while physical performance indicates severity. Sarcopenia assessment typically involves analyzing handgrip strength (HGS), muscle mass, and physical performance [3].

Muscle weakness often precedes muscle wasting in aging and other related diseases [2, 4], making it a valuable tool for predicting and monitoring disease progression. Studies have shown that muscle weakness is a stronger predictor of survival and disability than muscle wasting in old age [5]. Recognizing the prognostic significance of muscle weakness, the EWGSOP considers a low HGS as a sign of probable sarcopenia [3].

Due to the high cost and specialized personnel required for comprehensive diagnostic testing, the identification of suitable biomarkers or indices is crucial for the diagnosis and management of sarcopenia [6]. Such diagnostic indices are also relevant in non-cooperative, comatose, and/or bedridden patients, where traditional diagnostic measures, such as HGS assessment, body scans for muscle mass evaluation, and gait speed, cannot be measured.

Cystatin-c is a small protein produced by all nucleated cells. Its production rate remains stable and is minimally influenced by the alterations in muscle mass [7]. Previous studies have used a combination of plasma cystatin-c and creatinine levels for diagnosing sarcopenia [7]. However, most relevant studies involve hospitalized patients with various diseases [8, 9]. Conversely, the combination of cystatin-c and creatinine exhibits poor efficiency in diagnosing muscle wasting and sarcopenia phenotype in community dweller geriatric population [10]. Therefore, it may be imperative to investigate other biomarkers in combination with cystatin-c to predict the future onset of sarcopenia.

Sarcopenia is frequently associated with metabolic derangements and disrupted plasma lipid profiles [11]. Most studies investigating the association of plasma lipid profile with sarcopenia focus on low-density lipoprotein (LDL) cholesterols [12, 13]. Conversely, the prognostic relevance of total cholesterols to sarcopenia remains partly understood. A meta-analysis investigated 803,022 men and women aged 65–80, primarily from Asian countries [13]. The study found that sarcopenic patients had higher plasma total cholesterol levels than non-sarcopenic controls. However, these patients may also take lipid-lowering medications, which worsen sarcopenia by damaging skeletal muscle [14]. Conversely, low plasma total cholesterols may also be associated with sarcopenia status. This is partly because malnutrition is a common cause of sarcopenia [15], and malnourished older adults exhibit lower levels of plasma cholesterol than the age-matched controls [16].

However, the prognostic accuracy of total cholesterol in combination with cystatin-c remains elusive.

Most studies investigating the prognostic potential of plasma biomarkers for sarcopenia are conducted in hospitalized patients with specific diseases. In addition, most such studies are cross-sectional and do not establish causality. These studies also primarily investigate muscle mass with relatively less focus on muscle strength. Given that muscle weakness is considered a preliminary sign of sarcopenia [3], studies are required to investigate HGS with relevance to cystatin-c. However, a large-scale longitudinal study on community dwellers remains elusive.

We investigated the efficacy of the ratio of cystatin c and total cholesterol (CT ratio) in predicting low HGS among European older adults using the standardized Survey of Health, Ageing, and Retirement in Europe (SHARE) dataset [17]. We hypothesized that the CT ratio exhibits efficacy in predicting the future onset of low HGS in the European geriatric population. Specifically, we hypothesize that the CT ratio can effectively forecast the likelihood of an individual developing a low HGS. Furthermore, we explored how factors such as age, gender, quality of life (QoL), and specific comorbidities may influence the accuracy of this prediction. This research aims to contribute to the early identification and prevention of low HGS in this population.

## Materials and methods

The applied datasets stem from waves 6, 7, 8, and 9 of the SHARE survey, a representative multi-disciplinary panel study of individuals aged 50 and older [18]. HGS was measured using a hand-held dynamometer (Smedley, S Dynamometer, TTM, Tokyo, 100 kg), as described previously [19]. Participants were instructed to press the dynamometer twice with each hand. If one hand was affected, only the other hand was tested. During the test, the participants were asked to stand upright with their upper arm parallel to their body and their lower arm at a 90-degree angle. If needed, they could also perform the test seated. The highest of the four measurements was used for analysis. Individuals with swelling, inflammation, severe pain, recent injuries, or hand surgery were excluded from the study [19]. Similarly, the patients with kidney failure were excluded due to elevated cystatin-c levels [20]. The threshold for a low HGS was based on the guidelines by the European Working Group on Sarcopenia in Older People (EWGSOP2), as ≤ 27 kg for men and ≤ 16 kg for women [3].

All covariates were derived from the SHARE wave 6 as a baseline. Quality of life was assessed using the control, autonomy, self-realization, and pleasure (CASP-12) index [21]. This 12-item index consists of four subcategories with three questions each. Respondents rated how often they experienced specific feelings or thoughts on a scale of 1 (often) to 4 (never). The CASP-12 composite index, calculated by summing the scores of all 12 items, ranges from 12 (lowest well-being) to 48 (highest well-being). For analysis, the index was categorized into three groups: low (12–24), medium (25–36), and high (37–48) well-being [21].

Mental health was evaluated using the Euro-D depression scale, a 12-item index measuring the severity of depressive symptoms. Scores on this scale range from 0 to 12, with higher scores indicating more severe depression. For analysis, Euro-D scores were categorized into four groups: 0 (no depression), 1–3, 4–6, and 7–12 (highest level of depression) [19, 21].

Detailed questionnaires and self-reported data were used to obtain information about demography, BMI, lifestyle, and various diseases. These include difficulties performing multiple activities of daily living and several age-related diseases. The data about blood cystatin-c and total cholesterol levels was obtained from SHARE wave 6. The measurements involve the analysis of up to five dried blood spots from each of the 17,698 participants at their residences in 12 SHARE countries [22]. Only the participants with normal HGS in the baseline wave 6 were included in this study.

Cases with missing information about diseases or health conditions were assumed not to have the disease, e.g., only people with explicitly expressed diseases were counted as patients, which was valid for: difficulty with climbing several flights of stairs, difficulty getting up from a chair, difficulty getting dressed, bothered by frailty, falling down, high blood pressure, diabetes or high blood sugar, cancer, Alzheimer’s disease, stroke, and osteoarthritis. For BMI, Quality of life, and the Euro depression scale, we created a separate category representing missing information.

### Statistical analysis

We used multiple regression analyses to identify individual characteristics affecting the risk of low HGS. In the statistical approach, time *t* (years) to low HGS was modelled as

$${\text{In}}\,t = {\beta _0} + {\beta _1}{x_1} + {\beta _2}{x_2} + \cdots + {\beta _k}{x_k} + z$$

where *x*_1_,…, *x*_*k*_ represents the control variables, including the Cystatin-C/Total cholesterol ratio, gender, age, as well as mental and physical health, while *β*_1_,…, *β*_*k*_ is the effect of these individual characteristics. The *z* term is the error following the *f*(.) distribution with an extreme-value density yielding the Weibull regression model (and exponential model). The Weibull survival distribution was chosen due to its versatility, but the exponential and Gompertz survival distributions gave practically the same results (available from authors upon request). The hazard (empirically, probability of low HGS at time *t*, given normal HGS till time *t*) and survival (empirically, the probability of normal HGS at time *t*) functions are

$$h\left( t \right) = p \cdot \lambda \cdot {t^{p - 1}}\,{\text{and}}\,S\left( t \right) = {e^{ - \lambda \cdot {t^p}}}$$

with the parametrization

$$\lambda = {e^{ - p \cdot x\beta }}$$

where the shape parameter *p* is estimated from the data (*σ* = 1/*p*). Maximum likelihood is used to estimate the regression parameters by applying the STATA command STREG with a Weibull distribution (streg ${x}, distribution(weibull)). Based on the estimated regression parameters, hazard ratios (*h* in the second above equation) as well as the time to the occurrence (*t* in the first above equation) of a disease were predicted using the STATA command MARGINS (margins, at (CT_ratio = (25(10)105)) at means predict(hr)). The “margins” are statistics calculated from predictions of the fitted model at fixed values of the Cystatin-C/Total cholesterol ratio while averaging over the remaining variables. These margins are plotted into graphs.

The STATA software package 18.0 SE Standard Edition was used for the statistical analysis (Stata Statistical Software: Release 18. College Station, TX: StataCorp LLC).

## Results

At baseline in wave 6, 17,698 participants, including 7,722 men and 9,976 women, had normal HGS (Table 1). Among those, 8.1% of participants developed a low HGS during waves 7, 8, or 9. At baseline, the average cystatin-c levels were 0.978 mg/l, the average total cholesterol was 223.9 mg/dl, and the CT ratio was 44.1 (after multiplying with 10,000). The CT ratio was higher among men than women (Table 1). We found an age-dependent increase in the CT ratio from 50 to 60 to 90 + age groups. Similarly, gradually increasing BMI and lower scores on CASP-12 were also associated with a higher CT ratio. Next, we observed that higher scores on the Euro-D depression scale were associated with higher CT ratios. Similarly, participants with difficulties climbing stairs, getting up from a chair, and getting dressed also exhibited higher CT ratios. Lastly, the participants with high blood pressure, diabetes mellitus, cancer, Alzheimer’s disease, stroke, and osteoarthritis exhibited higher CT ratios than the participants without these diseases (Table 1).

**Table 1:** Basic characteristics of the study participants with normal HGS at the baseline in wave 6. (HGS; handgrip strength, BMI; body mass index)

Characteristic	Level	% with low HGS	Cystatin-C (mg/l) terol (mg/dl)	Total choles-	10,000×Cystatin / Cholesterol ratio	Sample size (*n*)
Gender	Male	8.2	0.983	220.3	45.1	7722
	Female	8.0	0.974	226.7	43.4	9976
Age	50–60	2.3	0.938	227.4	41.6	4092
	60–69	3.9	0.961	224.7	43.2	7077
	70–79	12.3	1.006	221.5	45.9	4956
	80–89	26.8	1.068	219.0	49.2	1493
	90+	52.5	1.132	223.5	51.3	80
BMI	Underweight	16.8	0.961	231.8	41.8	101
	Normal	7.2	0.964	227.2	42.9	6235
	Overweight	7.9	0.977	223.3	44.2	7305
	Obese	8.4	1.000	220.0	45.9	3811
	Missing	23.2	1.008	216.4	47.1	246
Quality of life	Low	16.4	1.002	219.5	46.1	304
	Medium	12.4	0.988	221.1	45.1	5177
	High	5.8	0.973	225.3	43.6	11,867
	Missing	13.7	0.996	221.8	45.4	350
Euro depression scale	No depression	6.4	0.969	223.8	43.7	3971
	Low	7.0	0.977	224.4	44.0	9620
	Medium	11.3	0.987	223.6	44.6	3318
	High	15.6	0.994	220.0	45.7	694
	Missing	12.6	0.980	223.7	44.1	95
Diff. with climbing several flights of stairs	No	5.9	0.968	224.8	43.5	14,043
	Yes	16.5	1.016	220.5	46.5	3655
Difficulty getting up from chair	No	7.0	0.973	224.3	43.8	14,781
	Yes	13.6	1.002	222.2	45.6	2917
Difficulty getting dressed	No	7.6	0.975	224.1	44.0	16,764
	Yes	16.3	1.024	221.4	46.7	934
Bothered by frailty, falling down	No	7.6	0.976	224.0	44.0	16,793
	Yes	17.0	1.015	222.3	46.1	905
High blood pressure	No	6.2	0.962	226.0	43.0	10,527
	Yes	10.8	1.002	220.9	45.8	7171
Diabetes or high blood sugar	No	7.4	0.973	225.3	43.6	15,456
	Yes	12.8	1.015	214.3	47.8	2242
Cancer	No	8.0	0.977	224.0	44.1	17,060
	Yes	10.7	0.991	221.7	45.1	638
Alzheimer’s disease	No	8.0	0.978	223.9	44.1	17,590
	Yes	19.4	1.017	223.2	45.9	108
Stroke	No	7.9	0.977	224.1	44.0	17,237
	Yes	13.7	1.027	218.1	47.5	461
Osteoarthritis	No	7.5	0.974	223.7	44.0	14,342
	Yes	10.6	0.994	224.9	44.6	3356
Total		8.1	0.978	223.9	44.1	17,698

Next, we investigated the correlations of low HGS with CT ratios in the study population (Table 2). We found statistically significant and robust correlations between HGS and CT ratios for both genders, age groups of 60–69 and 70–79, various BMI categories, and the low, medium, and high scores on the CASP-12 quality of life scale (Table 2). We also found significant correlations of low HGS with CT ratio based on the categorization of the study participants according to difficulty climbing stairs, getting up from a chair, or getting dressed. Lastly, the participants without high blood pressure, diabetes, cancer, Alzheimer’s disease, and stroke exhibited higher correlations between low HGS and CT ratio than the participants with these diseases (Table 2).

**Table 2:** Pearson’s correlations of Cystatin-C/total cholesterol ratio at wave 6 with the development of low HGS in waves 7, 8, and 9 in the study population (*n* = 17,698). (HGS; handgrip strength, BMI; body mass index)

Characteristic	Level	Correlation Coefficient	95% CI		*P* Value
			Lower	Upper	
Gender	Male	0.169	0.148	0.191	< 0.001
	Female	0.143	0.124	0.162	< 0.001
Age	50–60	-0.016	-0.047	0.015	0.000
	60–69	0.081	0.057	0.104	< 0.001
	70–79	0.092	0.064	0.120	< 0.001
	80–89	0.048	-0.003	0.098	0.000
	90+	0.125	-0.097	0.336	0.000
BMI	Underweight	0.232	0.038	0.409	0.000
	Normal	0.182	0.158	0.206	< 0.001
	Overweight	0.147	0.125	0.170	< 0.001
	Obese	0.118	0.087	0.149	< 0.001
	Missing	0.129	0.004	0.250	0.000
Quality of life	Low	0.158	0.046	0.266	0.000
	Medium	0.130	0.103	0.156	< 0.001
	High	0.147	0.130	0.165	< 0.001
	Missing	0.222	0.120	0.320	< 0.001
Euro depression scale	No depression	0.143	0.112	0.173	< 0.001
	Low	0.148	0.128	0.167	< 0.001
	Medium	0.162	0.129	0.195	< 0.001
	High	0.114	0.040	0.187	< 0.001
	Missing	0.404	0.220	0.560	< 0.001
Diff. with climbing several flights of stairs	No	0.127	0.111	0.143	< 0.001
	Yes	0.132	0.100	0.164	< 0.001
Difficulty getting up from chair	No	0.141	0.125	0.157	< 0.001
	Yes	0.165	0.130	0.200	< 0.001
Difficulty getting dressed	No	0.146	0.132	0.161	< 0.001
	Yes	0.170	0.107	0.231	< 0.001
Bothered by frailty, falling down	No	0.153	0.138	0.168	< 0.001
	Yes	0.112	0.048	0.176	< 0.001
High blood pressure	No	0.162	0.143	0.181	< 0.001
	Yes	0.119	0.096	0.142	< 0.001
Diabetes or high blood sugar	No	0.152	0.137	0.167	< 0.001
	Yes	0.109	0.068	0.150	< 0.001
Cancer	No	0.155	0.140	0.170	< 0.001
	Yes	0.124	0.047	0.200	0.000
Alzheimer’s disease	No	0.157	0.142	0.171	< 0.001
	Yes	-0.191	-0.367	-0.002	0.000
Stroke	No	0.157	0.142	0.171	< 0.001
	Yes	0.029	-0.063	0.120	0.000
Osteoarthritis	No	0.152	0.136	0.168	< 0.001
	Yes	0.157	0.124	0.190	< 0.001

As discussed above, only 8.1% of the study participants developed a low HGS during waves 7, 8, and 9. However, this incidence may be underestimated because many participants may likely develop a low HGS after wave 9. Thus, the traditional standard regression techniques are inadequate to handle the underestimation of this dataset. Therefore, we performed a survival analysis to predict the incidence of low HGS in the future (Table 3). We found that the female gender was associated with a 14% (parameter *β* = 0.861, *p* = 0.010) lower risk of developing a low HGS in the future. Similarly, the risk of developing a low HGS was increased with advancing age. For example, the risk was 51.3% higher (*β* = 1.513, *p* = 0.001) in 60–69 years old than in 50-59-year-old participants. Similarly, the participants aged 90 or above exhibited a 24 times higher risk (*β* = 23.545, *p* = 0.000) of developing a low HGS than the 50-59-year-old participants (Table 3). Conversely, higher scores on CASP-12 and the Euro-D depression scales were not associated with the risk of developing low HGS. Next, difficulty climbing stairs was associated with a 62.5% higher risk (*β* = 1.625, *p* = 0.000) of developing low HGS. Lastly, difficulty getting dressed (*β* = 1.214, *p* = 0.039), the presence of high blood pressure (*β* = 1.161, *p* = 0.009), diabetes mellitus (*β* = 1.173, *p* = 0.022), and osteoarthritis (*β* = 1.143, *p* = 0.043), were also associated with a significant risk of developing low HGS (Table 3).

**Table 3:** Parametric survival-time regression of the study participants showing the hazard rates of developing low HGS using the Weibull distribution. All the characteristics are from wave 6 and are controlled for the country using the Cox proportional hazard model (*n* = 17,690). (HGS; handgrip strength, BMI; body mass index). (being a female lowers the risk of low HGS by 0.87 (13% lower than males), independent of Cystatin and cholesterol ratio)

Characteristic	Level	Hazard rate	*P* value	95% CI	
				Lower	Upper
Cystatin-C/total cholesterol ratio		1.013	0.000	1.006	1.020
Female		0.861	0.010	0.769	0.965
Age	60–69	1.513	0.001	1.197	1.911
	70–79	4.281	0.000	3.431	5.343
	80–89	8.633	0.000	6.815	10.935
	90+	23.545	0.000	16.040	34.563
BMI	Normal	0.455	0.002	0.279	0.742
	Overweight	0.429	0.001	0.263	0.699
	Obese	0.401	0.000	0.244	0.659
	Missing	0.579	0.053	0.333	1.008
Quality of life	Medium	1.181	0.283	0.872	1.599
	High	0.914	0.585	0.663	1.261
	Missing	1.005	0.981	0.662	1.526
Euro depression scale	Low	0.899	0.156	0.775	1.042
	Medium	1.067	0.468	0.895	1.273
	High	1.175	0.219	0.909	1.519
	Missing	1.534	0.159	0.846	2.783
Diff. with climbing several flights of stairs		1.625	0.000	1.432	1.845
Difficulty getting up from chair		1.137	0.064	0.993	1.303
Difficulty getting dressed		1.214	0.039	1.009	1.460
Bothered by frailty, falling down		1.159	0.101	0.972	1.382
High blood pressure		1.161	0.009	1.039	1.298
Diabetes or high blood sugar		1.173	0.022	1.023	1.345
Cancer		1.033	0.795	0.808	1.321
Alzheimer’s disease		1.073	0.753	0.690	1.668
Stroke		0.998	0.987	0.769	1.294
Osteoarthritis		1.143	0.043	1.005	1.301
Constant		0.003	0.000	0.0013	0.0056
/ln_p		0.652	0.000	0.6015	0.7015
*p*		1.918	0.000	1.8249	2.0169
1/p		0.521	0.000	0.4958	0.5480

**Fig. 1:**
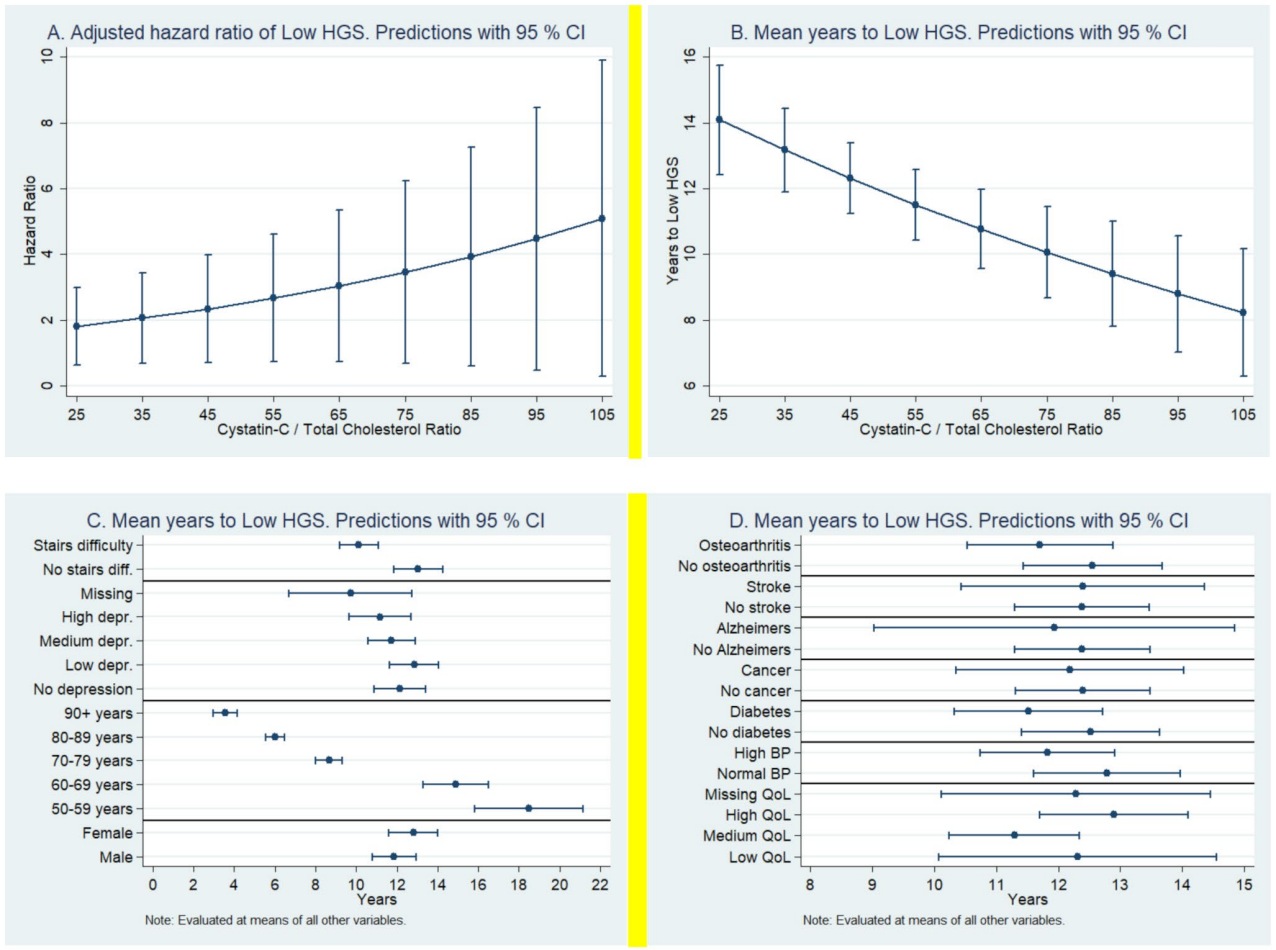
Hazard ratios of developing low HSG (**A**) and average number of years required to develop low HGS based on cystatin-c/total cholesterol ratio (**B**), difficulty climbing stairs, depression, age, and gender (**C**), and various comorbidities and poor quality of life (**D**) in the study participants (*n* = 17,698). (HGS; handgrip strength, QoL; quality of life)

Lastly, based on the regression coefficient values presented in Table 3, we performed various stimulations to investigate the associations of CT ratios with the risk of developing low HGS, the mean number of years required to develop low HGS based on CT ratio, various comorbidities and demographic factors (Fig. 1). We found a positive correlation between the CT ratio and the hazard of developing low HGS. For example, a CT ratio of 35 was associated with a hazard ratio of 2.1 for developing low HGS (Fig. 1A). Similarly, the CT ratio of 105 was associated with a hazard ratio of 5.1 for developing a low HGS. Next, we transformed the hazard ratios to the average years required to develop a low HGS (Fig. 1B). We found a negative association between the CT ratio and the average number of years required to develop low HGS. For example, the participants with a CT ratio of 25 required 14.1 years to develop low HGS. On the other hand, participants with a CT ratio of 105 required only 8.2 years to develop low HGS (Fig. 1B). We also investigated the associations of demographic factors and comorbidities with the number of years required to develop low HGS (Fig. 1C). After adjusting for other variables, women required nearly one year more to develop low HGS than men. Advancing age was associated with a lower time to develop low HGS. Lastly, higher depression and difficulty climbing stairs were associated with lower time to develop low HGS (Fig. 1C). Lastly, among multiple comorbidities, the presence of Alzheimer’s disease was associated with less time to develop a low HGS (Fig. 1D).

## Discussion

We report significant efficacy of CT ratio for probable sarcopenia in 17,960 community-dwelling older adults from 12 European countries. Specifically, the increasing CT ratio was associated with a higher risk and earlier onset of low HGS in the study population. We also identified advancing age, male gender, higher than normal BMI, a low QoL, depression, and difficulties climbing stairs, rising from a chair, and getting dressed as the factors that strengthened the prognostic efficacy of the CT ratio for probable sarcopenia. Lastly, we also found that advancing age, male gender, difficulties climbing stairs, rising from a chair, and dressing up, and the presence of depression, high blood pressure, Alzheimer’s disease, and osteoarthritis increased the risk of developing low HGS.

This is the first large-scale study reporting the prognostic efficacy of the CT ratio for probable sarcopenia. Previous studies have reported the predictive accuracy of cystatin-c with other biomarkers for muscle weakness and wasting [8, 9]. However, such findings were reserved for small subsets of hospitalized patients and were not replicated in community-dweller older adults [10]. We investigated a large community-dwelling population in residential settings, which is relevant to several age-related lifestyle factors and comorbidities.

We observed significant correlations between the CT ratio and handgrip strength in both men and women, regardless of BMI. This supports the general applicability of our findings across different segments of the population. The correlations were more robust in men than women of all age groups. From the sixth decade onward, men develop a steeper decline in HGS than women, with a higher relevant risk threshold for mortality [23]. HGS and CT ratios also exhibited stronger correlations in both genders during the seventh and eighth decades of life. However, similar observations were not found among the participants aged above 80. While this observation seems counterintuitive, a significant decline in plasma cystatin-c levels is reported from the middle of the eighth decade of life [24]. Similarly, a gradual reduction in total cholesterol levels is also observed during the eighth decade of life and later [25]. This decline may uncouple the cystatin-c and total cholesterol levels from HGS in advanced age. Interestingly, the predictive efficacy of total cholesterol for mortality also reduces with advancing age [25]. Thus, it appears that various comorbidities and lifestyle factors weaken the associations of total cholesterol with mortality and relevant diseases, including sarcopenia. Lastly, the data from participants of advanced age should be cautiously interpreted due to their selective survival.

A low or medium score on the CASP-12 QoL scale was associated with stronger associations between CT ratio and HGS. It is previously reported that lower scores on CASP-12 are associated with lower HGS in older adults [26]. We found lower CASP-12 scores were associated with the strongest correlation between CT ratio and HGS, compared to medium and high scores. An inverse association between depressive symptoms and HGS has also been recognized [27]. We found a higher risk of developing low HGS in participants with higher depression. Additionally, the regression values between the CT ratio and HGS were consistent across three Euro-D categories of depression, with slightly higher regression for participants with medium and high depression. The CT ratio appears to be a better predictor of outcomes for low handgrip strength in older adults who also experience poor quality of life and higher levels of depression. Both poor quality of life and depression are independent risk factors for low handgrip strength, and their presence seems to amplify the predictive power of the CT ratio.

We have previously reported that difficulties performing activities of daily living, such as climbing stairs, rising from a chair, and getting dressed, are associated with low HGS [19]. This observation reflects the generalized sarcopenia process in whole-body muscles. Consistent with these reports, we found that the risk of developing low HGS was higher in older adults with difficulties performing these activities. We found consistently robust and significant regressions between CT ratio and HGS in all study participants with or without difficulties performing these activities. Additionally, the participants with difficulty getting up from a chair or being dressed exhibited further strengthening of the relation between CT ratio and HGS. However, similar observations were not found in participants with difficulty climbing several flights of stairs. Thus, the prognostic efficacy of the CT ratio for low HGS is not reduced by difficulties performing routine activities of daily living.

As expected, various comorbidities, including high blood pressure, diabetes mellitus, Alzheimer’s disease, and osteoarthritis, were associated with a higher risk of developing low HGS. These comorbidities accelerate the degenerative processes in the skeletal muscle, which leads to the worsening of HGS [28]. The CT ratio exhibited significant prognostic efficacy for low HGS in all participants, irrespective of the comorbidities. While the CT ratio predicted low handgrip strength in all study participants, its predictive power was stronger in participants without comorbidities compared to those with comorbidities. However, the participants without comorbidities exhibited higher prognostic efficacies for low HGS than those with comorbidities. Thus, it appears that comorbidities reduce the prognostic efficacy of the CT ratio for low HGS. The myotoxic effects of these comorbidities may involve mechanisms independent of plasma cystatin-c and total cholesterols. For example, diabetes mellitus causes glycosylation of muscle contractile apparatus with adverse effects on muscle force-generating capacity [29]. Similarly, Alzheimer’s disease causes muscle weakness by affecting peripheral motor neurons and neuromuscular junction [30]. Our data shows that such mechanisms may operate independently of plasma cystatin-c and total cholesterol.

This study demonstrates several major strengths. We investigated a large, representative sample drawn from 12 European countries. The longitudinal design significantly bolsters our confidence in the predictive efficacy of the CT ratio for low HGS. The standardized SHARE questionnaire, administered consistently across multiple European settings, ensured data harmonization and enhanced the study’s reliability [18]. The study’s limitations include the potential for undetected subclinical illnesses among participants during assessment. Additionally, mild to moderate heterogeneity across European countries may have influenced our findings. The presence of multiple comorbidities in some participants could have had a more pronounced effect on our observations than the cumulative impact of individual comorbidities. We did not measure body fat content, which can adversely affect muscle strength [31] and, subsequently, the association between CT ratio and low HGS. The measurements of BMI may not accurately represent body fat content. Aging and co-morbidities are associated with systemic inflammation [32], which can contribute to the sarcopenia process [33]. However, we did not measure the levels of inflammatory cytokines in the study population. Finally, selective survival among study participants may have introduced bias into our data. This limitation is particularly important to consider when examining participants aged 90 and older. This group has a greater life expectancy than the European average of 82 years for women and 75 years for men [34]. Therefore, caution is advised when extrapolating our findings to larger populations.

In conclusion, we report that the measurement of the CT ratio may be a useful tool to predict low HGS in older adults. Gender, advancing age, QoL, depression, difficulties performing activities of daily living, and various comorbidities do not significantly affect the prognostic efficacy of the CT ratio for low HGS. Our findings have clinical and policy implications, potentially aiding healthcare providers and policymakers in identifying older adults with muscle weakness.

## Data Availability

The data is publicly available after application from https://share-eric.eu/. The access to data requires an individual free registration followed by the acceptance of the SHARE Conditions and signing the SHARE User Statement. After acceptance of these documents, data can be downloaded using the personal ID and password.
